# Barriers and Facilitators to Pediatric Resident Education in the Emergency Department: A Qualitative Study

**DOI:** 10.7759/cureus.40142

**Published:** 2023-06-08

**Authors:** Christopher Jones, Jennifer Mitzman, Sandra Spencer, Charmaine B Lo, John D Mahan, David Stein

**Affiliations:** 1 Emergency Medicine, Nationwide Children's Hospital, Columbus, USA; 2 Emergency Medicine, Colorado Children's Hospital, Aurora, USA; 3 Pediatric Nephrology, Nationwide Children's Hospital, The Ohio State University, Columbus, USA; 4 Educational Studies, The Ohio State University College of Education and Human Ecology, Columbus, USA

**Keywords:** facilitators, barriers, emergency department, qualitative analysis, resident education

## Abstract

Objective

Local resident evaluations of the pediatric emergency department (ED) declined over the last five years. Sparse literature exists on resident perspectives of educational experiences. This study explored the barriers and facilitators to resident education in the Pediatric ED.

Methods

This qualitative study utilized focus groups at a large pediatric training hospital. Trained facilitators performed semi-structured interviews prompting discussion of resident experiences in the pediatric ED. One pilot and six focus groups (38 pediatric residents) achieved data saturation. Sessions were audio recorded, de-identified and transcribed by a professional service. Three authors (CJ, JM, SS) analyzed the transcripts independently using line-by-line coding. Following code agreement, authors identified central themes drawing on grounded theory.

Results

Six categories emerged: (1) ED environment, (2) consistent goals, expectations, and resources, (3) ED workflow, (4) preceptor accessibility, (5) resident growth and development, (6) ED preconceived notions. Residents value a respectful work environment despite the chaotic nature of the ED. They need clear goals and expectations with a strong orientation. Autonomy, open communication and shared decision-making allow residents to feel like team members. Residents gravitate toward welcoming, available preceptors that enthusiastically teach. More ED environment exposure increases comfort and efficiency and helps develop medical decision-making skills. Residents admit ED preconceptions and personality traits affect performance.

Conclusion

Residents self-identified barriers and facilitators to ED education. Educators must provide a safe and open learning environment, clear rotation expectations and goals, consistent positivity supporting shared decision making, and allow residents autonomy to build their practice styles.

## Introduction

The Accreditation Council for Graduate Medical Education (ACGME) mandates all pediatric residency programs to provide educational experiences to further resident learning [1-3]. The emergency medicine rotation is integral to these experiences. Recently, the emergency medicine rotation at a large pediatric residency noted a significant drop in resident satisfaction scores. After multiple faculty-driven attempts to improve these scores, educational leaders noted the resident perspective of what constitutes a good educational experience had not been explored. A literature review revealed few publications involving resident perspectives of their own education.

Education literature discusses resident learning style preferences, learning on rounds, noon conference preferences, and the association between educational experiences and in-training exam scores [4-17]. Some literature has focused on resident viewpoints of educational experiences [18-23]. A 2016 study of surgical residents found that the most learning occurred in clinical experiences involving evaluation and management, technical skills, complication identification and management, and teamwork [19]. A 2018 study of internal medicine residents found that inpatient residents learned from repetition, effective pedagogy, clinical problem-solving, opportunity for active engagement, and bedside learning [22].

While these studies have begun the transition to resident-centered educational experience, limited literature focuses on pediatric residents and none involve their educational experiences in the pediatric emergency department setting. Our goal was to determine whether the emergency department (ED) provides a good learning environment and to identify the barriers and facilitators to their education.

## Materials and methods

Ethical approval

IRB approval was obtained by NCH and this study was deemed exempt.

Sampling

We conducted a qualitative study using focus groups at a large pediatric academic hospital to discover the barriers and facilitators to resident education in the pediatric emergency department. The study occurred from October 2018 to December 2019. Participants were selected via a purposive sampling technique due to the expertise of the current pediatric residents [24]. All pediatric resident physicians in this residency program were deemed eligible. Exclusion criteria included any members of the internal medicine/pediatric combined residency and any residents who declined to be audio recorded.

Residents were contacted through direct e-mail by the ED research team with an enrollment letter (Appendix A) and a nominal incentive of a $5 gift card lottery and light refreshments were offered for participation. The ED research team is not involved in patient care or evaluation of the resident physicians. The authors of the study and ED faculty were blinded to resident participation, allowing for resident confidentiality.

Focus groups

Two members of the ED research team conducted the focus groups and utilized a semi-structured interview format (Appendix B) to discuss resident experiences in the emergency department [25]. The authors developed the questionnaire since there were no previous studies to draw upon for guidance. Focus groups took place in a private, easily accessible conference room. After completing a demographic sheet (Appendix C), residents were reminded they would be audio-recorded during the one-hour session, and verbal consent was obtained. Residents began the focus group by selecting an animal representation to support anonymity and simplify the subsequent transcription. Audio recording was done with a handheld recorder, which was stored in the facilitator's locked office.

Participants

Seven focus groups were performed from November 2018 to May 2019, totaling 38 of 109 eligible pediatric residents (Table 1).

**Table 1:** Participant Demographics (N=38)

		Mean	Median (IQR)
Age		28	28 (27-29)
		N	%
Gender	Female	32	84%
Race	White	30	79%
	Black/AA	1	3%
	Asian	5	13%
	South Asian	1	3%
	Biracial	1	3%
Marital Status	Single	21	55%
PGY Year	PGY1	10	26%
	PGY2	14	37%
	PGY3	14	37%
Planned Career Specialty	General Pediatrics	14	37%
	Acute Care Specialties (ICU/ER)	7	18%
	Other Subspecialties	14	37%
	Undecided	3	8%

The first group, comprised of pediatric residents, functioned as a pilot group, to confirm question clarity and session flow, but was not analyzed [26]. Subsequently, six focus groups were held ranging in size from 4-10 participants. A professional transcription service transcribed the audio recordings.

Analysis

Three of the authors (CJ, JM, SS) analyzed the transcripts independently using line-by-line coding and constant comparative method [27]. Each transcript was individually coded by two members with the third member available to resolve any disagreements. After code book creation and agreement, authors identified central themes drawing on grounded theory. Data saturation was achieved by the 6th focus group analysis and further focus group enrollment was discontinued.

## Results

Focus group analysis identified six central themes: ED environment, preconceived notions, consistency in goals, expectations and resources, ED workflow, preceptor accessibility, and resident growth and development. Table 2 highlights pertinent quotations from participants supporting each theme.

**Table 2:** Quotes Representing Focus Group Perceptions of Barriers and Facilitators

Theme	Representative quotations
Emergency Department Environment	
Unpredictability and chaotic environment leads to unwanted stress and anxiety	"When you're learning you need a second or two to kind of take everything in...and not have somebody jump in and take over right away because then, I never get over that deer in the headlights thing."_ (Badger, 3.19)_
Undifferentiated patients offer a unique educational experience	"...you get these undifferentiated patients... I want to order X,Y, and Z labs because I'm looking for these things and I think that's simply something we don't get except for an urgent care." _(Dolphin, 4.19)_
Supportive ancillary staff promote a psychoemotional safe learning space for trainees	"An environment where you feel safe not knowing things is really important” _(Elephant, 5.19)_ “I've had some attendings…who very much make an environment of, it's okay to not have every answer and I've had some attendings that have done that very badly.” _(Elephant, 5.19)_
Opportunities to practice procedural skills strengthen resident skill sets	"I was on my procedure shift and there was a kid with a forehead laceration and I got to suture him. The suture tech walked me through everything, stayed in the room with me and made me feel super comfortable." _(Otter, 12B.18)_
Consistency in Goals, Expectations and Resources	
Formal, shared rotation goals and expectations facilitate learning	"Part of a good learning experience involves having clear learning objectives and a good plan...and some kind of format to meet those objectives, to have to get structured learning in an efficient way that you know you're going to get some kind of reliability within a rotation.” _(Panda, 5.19)_ "Having a more formal orientation as an intern and maybe even a brief one at the beginning of second year before you have those trauma shifts would be helpful so you know these are the expectations.” _(Elephant, 4.19)_
Consistent learning resources standardize experiences and strengthen a rotation	"NICU sends a very detailed orientation email that has lots of resources and many residents probably don't read it in its entirety after their intern year but it's there if you want it.” _(Turtle, 4.19)._
Emergency Department Workflow	
Shared decision-making allows residents to feel like valued members of the team	"I was a part of the medical decision-making team and my input had value and they were asking my ideas...which was really neat...because it's happened before where the Attending and Fellow make decisions and they don't include you at all, you're kind of the person putting in the orders."_(Eagle, 12B.19)_
Increasing continuity with preceptors increases trust, rapport, and graduated learner autonomy	”The attendings would be more comfortable with me maybe coming up with some of my own plans because they trusted me a little bit more as a second year...there was a bit more control, understanding, and respect between the two of us."_(Husky, 3.19)._
Increased providers in the ED allow for resident education to overcome service	"I get this sense from the staff that they are more concerned with the throughput than they are with our education...I think a lot of our frustration stems from that."_(Kangaroo, 2.19)_ "A setting where as a resident you are useful but not 100% essential to the clinical practice functioning...because then the burden of functionality interferes with your ability to learn or do something"_(Koala, 12A.18)_ "The ED is such a busy place...we're expected to take on more patients which I think our education kind of goes by the wayside...if it was possible to get more coverage...it would help our education all around."_(Koala, 3.19)_
Preceptor Accessibility	
Inconsistent availability of preceptors to staff patients creates a workflow dilemma	"One big frustration in the ED is waiting to staff, the attendings are busy, and we have to wait sometimes 20 minutes and then feel pressure to pick up more patients." _(Unicorn, 2.19)_ "If you're waiting to staff and you go to see another patient, because the attending is busy in a big trauma…when I came back to staff, I was told don't do that." _(Koala, 5.19)_
Preceptor approachability to staff patients provides a space where residents feel protected	"The ER is high acuity, busy, and chaotic...it builds high stress levels...but you chose this as your job and you chose to be in an academic institution with learners and therefore should be kinder, more patient, and more understanding because if you're not going to be, then why did you choose this as your job?" _(Elephant, 5.19)_
Preceptor’s desire to teach positively affects education	"One attending...pulled me aside and gave me feedback and also gave me an article that pertained to one of our patients...it made me feel good because he was thinking of my education and because I went home and read the article after my shift. _(Koala, 3.19)_
Resident Growth and Development	
Increasing ED exposure increases resident comfort and confidence	"The ER gets better as they progress in their training...it's the hardest as an intern...you get accustomed to the ER and feel more comfortable caring for critically ill patients and then by third year...you have a lot more people say I wasn't dreading my shifts, as much. _(Badger, 3.19)_ "When you've finished your third year you start to notice you can create a differential and workup...but it's not containing extraneous things that the intern differentials do and a more targeted workup to say this is what I want to do." _(Dolphin, 4.19)_
Hands on training improves pattern recognition and skill sets	"I feel successful when I see a patient at the beginning of the month and maybe I don't know what to do...then I see a similar patient at the end of the month and I feel comfortable managing them._ (Badger, 3.19)_ "One thing that will always stick with me...I saw my first new onset DKA undifferentiated patient...one of the nurses was in there when I was getting a history and was like...do you want a sugar? And I was like, oh good thought, let's do that. So now I will remember what these things truly look like because I hadn't seen them in person yet.”_ (Koala, 12A.18)_
Emergency Department Preconceived Notions	
Internalizing previous residents’ experiences clouds residents learning experiences	"There was a lot of negative talk about how the ED is run...a lot of negative perceptions amongst residents...that hindered my willingness to approach people." (Turtle, 4.19) "I had heard that it’s just a really difficult rotation and no matter how good of a resident you are, someone will always be upset at you...but I was pleasantly surprised. (Hippo, 5.19)
Residents’ personality characteristics and career interests affect their learning mindset	"The ER is always going to be a challenging place because it's busy...some people do not thrive in that kind of an environment, whereas there are others who don't thrive in a primary care office." (Dolphin, 4.19) "Part of it is…a lot of us are probably more perfectionist driven personalities...the negative things...stick with me a lot more than any of my positive experiences." (Husky, 3.19)

Emergency department environment

Resident physicians expressed that the ED is unlike many other training sites. It is busy, functionally chaotic, and particularly unpredictable. While unpredictability can lead to fear and anxiety, the presence of supportive teams counteracted those feelings. In addition, the fast pace often provides less time for trainees to process their patient encounter and offer a well-developed clinical plan. This differs from the experience on outpatient clinics and even inpatient wards. Positively, residents stated the ED inherently offers a unique experience with undifferentiated patients and the ability to see a patient from the start of their work-up often to diagnosis and disposition. Finally, the opportunity to practice a variety of procedures improves their skill set and confidence and facilitates their education.

Preconceived notions of the emergency department

Junior residents often ask senior residents for guidance on upcoming rotations. Many comments about the ED are negative, influencing junior residents' judgment prior to the rotation. Junior residents feel apprehensive in the ED setting and are often anxious about the upcoming interactions. Residents also state personality traits and career aspirations affect their performance. Those residents interested in acute care specialties find the ED challenging but rewarding, while residents desiring an outpatient career perceive the ED to be overwhelming and stressful.

Consistent goals, expectations, and resources

Rotation structure is important to residents. They value clear, consistent expectations with easily accessible references. The lack of availability of current, centralized references such as guidelines, quality improvement projects, seminal evidence-based articles, and protocols while rotating through the department, differs from other rotations. Furthermore, although the roles of junior and senior pediatric residents are distinct, there is a lack of role clarity on shift, particularly from intern to second year. Residents stated that improved orientation for pediatric residents could clarify these concerns.

Emergency department workflow

Participants recognize the need to balance patient safety and flow with resident education in the ED. The focus on departmental flow pressures residents to see more patients and focus on documentation, however, residents value learning opportunities that stem from inclusion in decision-making. Residents want to contribute and be considered valued team members, however when orders are placed prior to resident evaluation and care plans are independently developed by preceptors, residents feel excluded. Furthermore, residents stated that their infrequent experiences with the same faculty member may have impacted entrustment and rapport, which they feel decreased their autonomy in the ED.

Preceptor accessibility

Participants prefer approachable preceptors who teach, talk through cases and differentials, give clinical pearls, and offer feedback on exam findings. Residents value case discussions that create teaching points not found in textbook medicine. An experienced preceptor’s thought process reveals many aspects of the way ED doctors think and ways to avoid pitfalls.

Residents state that competing demands on their preceptors limit their availability for teaching and staffing new patients. Residents understand the urgency of more critical patients but do not feel empowered to start work-ups when staffing is unavailable.

Resident physician growth and development

While interns may initially feel lost and helpless in the ED, participants mentioned as they move through residency they become accustomed to the ED environment and their medical knowledge expands. They state that managing patients from initial presentation until diagnosis allows them to improve their differentials, work-ups, and pattern recognition and increases comfort and confidence in the ED. Residents can call upon previous encounters which allows them to see, present, and manage patients more efficiently and confidently. Thus, at the end of their experience, senior residents understand ED function and decision-making processes.

## Discussion

Educational research focusing on resident perspectives of their own education is lacking as most of the available literature has primarily addressed faculty and program directors [18-23]. The few resident perspective studies that have been conducted have focused on surgery and internal medicine education, there have been no studies exploring pediatric residents’ educational experiences, particularly in the Emergency Department [19,22]. The authors conducted this novel study to assess education in the Emergency Department from the pediatric trainee perspective. This study capitalizes on adult learning theory and utilizes the learners to self-identify gaps in their education. Knowles introduced the five (5) assumptions of adult education which include the learner’s desire to be: self-directed (1) and self-motivated (2) in their problem-centered learning (3), experientially driven (4), and ready to learn (5).

Through six themes, the participants expressed key drivers to their education. With the chaotic ED conditions (1), clear rotation expectations and goals (2) help to clarify their role in the educational experience. Despite preconceived rotation expectations (3) affecting their outlook, the residents stated through exposure to the ED environment they became more acclimated with the team dynamic, decision-making, and workflow (4) of the department which improved their overall confidence in patient care (5). Finally, easily accessible, supportive preceptors (6) guide residents through the undifferentiated patient and procedural opportunities which help resident education.

**Figure 1:**
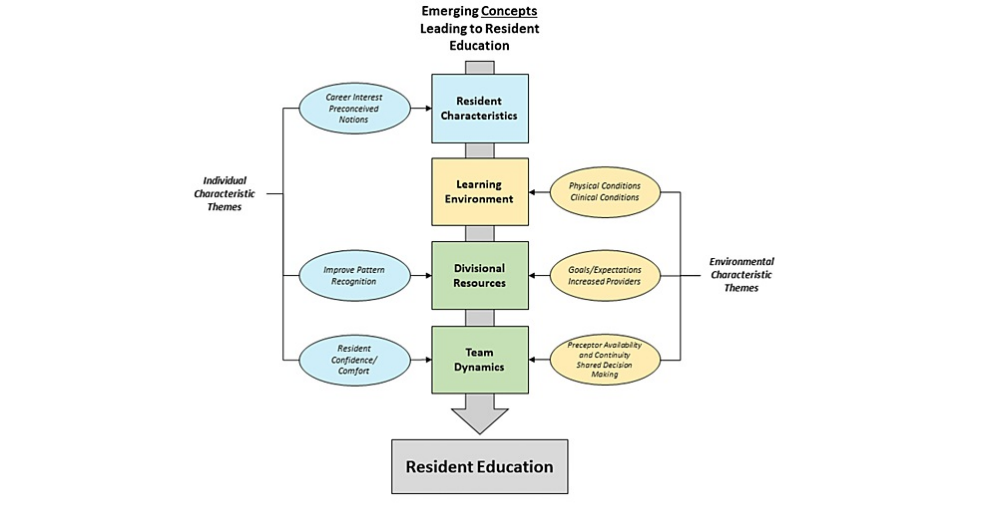
Concepts contributing to resident education

The authors determined that these themes can be viewed as both individually and environmentally focused characteristics. These characteristic themes contribute to broader concepts that contribute to resident education, including resident characteristics, learning environment, divisional resources, and team dynamics (Figure 1).

Resident characteristics

Participants suggest a resident’s personality affects their educational experience. Participants interested in ICU or ED fellowship, which were 18% of participants, had more positive experiences in the ED. Many residents did suggest that some residents inherently do not thrive in fast-paced environments. This correlates with Knowles’ self-motivated assumption, which suggests the need to refocus our educational efforts for primary care-focused residents. Discussions on what can be accomplished outpatient and when to refer to the ED may support their educational goals.

The residents suggest other trainees should approach each rotation formulating their own opinion based on experience, which is in line with Knowles’ ready-to-learn assumption. Keeping an open mind in each educational experience will provide residents the space to learn from each encounter. Having a negative attitude and preconceived dislike for a rotation may lead to a self-fulfilling prophecy. A negative critique by a senior resident may subconsciously bias junior residents against a rotation. One resident mentioned how negative perceptions kept them from approaching people in the ED and another stated they were pleasantly surprised after creating their own opinion of the rotation.

Learning environment

The learning opportunity provided by evaluating the undifferentiated patient emphasizes Knowles’ assumption of problem-centered learning. The undifferentiated patient provides the opportunity to build differentials, develop evaluations, practice procedures, communicate with consultants, balance multiple layers of patient care, and improve their efficiency. This unique offering is not found in other rotations.

A supportive learning environment allows residents to work through patient management and learn from their experiences which supports Knowles’ ready-to-learn and experiential learning assumptions. A psycho-emotionally safe learning environment permits residents to brainstorm and communicate with their patient care team about medical history, differentials, medical management, and reassessments. This supportive environment enhances the resident experience by opening a space for failure without ridicule and allows residents to strengthen differentials, narrow evaluations, perfect procedures, and ultimately move into confident physicians prepared for independent practice.

Divisional resources

Residents easily identified educational resources the ED could provide that align with Knowles’ self-directed and ready-to-learn assumptions. Residents desire clear, accessible objectives placed in a reference document. Some residents suggest delineated expectations based on residency program (Emergency Medicine, Pediatrics, Family Medicine) and level of training could help. Providing residents learning resources such as algorithms, clinical guidelines, evidence-based articles, and frequently referenced material allows residents to explore concepts associated with their patients. This will allow residents to focus on patient care during their shift and utilize the documents for clarification needs. These resources could further be emphasized during the in person and slide deck orientations provided at the start of the rotation.

Knowles’ ready-to-learn assumption is supported by residents’ recognition that more time to critically analyze patient encounters and spend less time with workflow tasks improves education. Residents comment that with the sheer volume of patients seen, increased provider presence would allow residents a decrease in service and focus on education. Additional advanced practice providers or physicians would help to remind faculty members that the resident physicians’ main function in the ED is not flow.

Team dynamics

Supportive preceptors who provide resident autonomy in the setting of seamless patient care allow residents to capitalize on all five of Knowles’ assumptions for adult learning. Preceptors who show enthusiasm for teaching, allow residents to create a plan, explain their clinical reasoning and evidence-based practices, and provide constructive feedback strengthen the resident educational experience. Residents also appreciate preceptors who keep them updated on patient status or plan changes allowing for team-based management decisions.

Increasing resident autonomy supports Knowles’ self-direction and self-motivation in learning assumptions. Preceptor accessibility was a universally cited concern. Physical availability of preceptors can be challenging. Increased preceptor continuity would build trust, rapport, and autonomy and inspire confidence in resident independent management when the preceptor is occupied with acutely ill patients. Residents would then feel empowered to move forward with plans and feel like a contributing member of the team.

ED team functionality affects resident clinical experiences. Increasing the ED exposure directly increases the resident’s comfort and confidence. As their medical knowledge grows, so does their confidence in patient encounters. This comfort in the ED supports Knowles’ experiential learning assumption which allows residents to more seamlessly present assessment and plans and initiate work-ups both independently and with their preceptors.

## Conclusions

Effective resident education in the ED is built on the concepts of resident characteristics, learning environment, divisional resources, and team dynamics. Participants identified themes that affect these educational concepts and support Knowles’ five assumptions of adult learning. As self-directed learners, residents desire consistent goals, educational resources, and increased decision-making autonomy. Residents become more self-motivated when the chaotic ED environment is tailored to their own educational goals and applicability for their future career aspirations. Starting their rotation with an open mind and entering a supportive learning environment that allows time for building rapport with supportive preceptors and processing patient management decisions allows residents to be ready to learn. Trainees can utilize the unique learning opportunities of the undifferentiated patients in the fast-paced ED to work on problem-centered learning. Finally, as their medical knowledge and patient encounters expand, residents will become more comfortable and confident utilizing their experiential learning to prepare for independent practice. By enhancing facilitators and removing the barriers linked to these assumptions, the ED will become a more educationally rich learning environment.

## Appendices

APPENDIX A: Welcome Letter

Dear Pediatric Residents,

            My name is Christopher Jones and I am a 2nd year Pediatric Emergency Medicine Fellow here at Nationwide Children's Hospital. I am currently working on a project to determine if the emergency room provides a good learning environment for Pediatric residents and to identify barriers or facilitators to your education in the emergency room. I am inviting you to participate in a focus group to help us understand the needs of the pediatric residents.  

            Focus groups will occur on campus and will last between 1-2 hours. Focus groups will be facilitated, recorded, and then anonymously transcribed by research staff who have no influence on your ER rotation/grade. We will have food and light beverages for your time and commitment to this project.

            If you are interested, please follow the link at the bottom of this email to sign up for focus group times. Please select as many possible groups that you can attend. Our research team will reach out to you with the selected date/time of your focus group.

            Your opinions truly matter to us! We hope to create a quality improvement project involving your ER rotation with the data we collect.

            Feel free to reach out to me (), Jennifer Mitzman (), or Sandra Spencer () with any questions you may have about the project.

Doodle Poll Link here.

Thanks for your consistent hard work,

Christopher Jones, DO

APPENDIX B: Semi-Structured Interview Questions

            Opening: Tell us your "animal" representation and your favorite vacation destination

            Introduction: When you hear the words resident education/successful rotation, what comes to mind

            Transition: What were your first impressions of the ER.

            Key: What are you hearing people say about the ED rotation in the residency

            Transition: Think back to your experiences in the Emergency Room. Tell me about one that made a lasting impression and how did it make you feel?

            Key: What part of that experience made you feel that way? 

            Key: Of the negative experiences, what could have been changed to make it better?

            Key: If you could change one thing about your ED rotation, what would you change? AND what's the main reason that thing needs changing

            Transition: What does your residency program do to provide education in the ED?

            Key: If you were in charge of resident education in the ED, what kind of changes would you make? 

            Key: What additions to the ED would advance your education? 

            Key: What do ERs of the future need to be like to ensure the success of all learners? Describe the ER setup

            Key: Of all of the changes you have mentioned, what is most important to you?

            Optional Activity for last key: In this activity we have discussed barriers and facilitators quite a bit. As a group, what do you believe is the Number 1 most important barrier to a successful education in the ED? What is the number 1 most important facilitator?”

            End: We are trying to make your ED rotation one of the best. What advice do you have for us?
 

APPENDIX C: Demographic Questionnaire

1) What is your gender?

            a) Female  b) Male  c) Nonbinary/Third gender d) Prefer to self describe as ___

      e) Prefer not to answer                      

2) Please provide your current age.

3) What is your current postgraduate year?

            a) PGY1 b) PGY2 c) PGY3 d) PGY4/chief

4) What is your marital status?

            a) Single, never married b) Married c) Divorced d) Separated e) Widowed

5) What is your self-identified race?

            a) White b) Black or African-American c) American Indian or Alaskan Native

            d) Asian e) South Asian f) Pacific Islander g) Some other race.

6) What is your planned future Pediatric specialty?

PGY-5

Pediatric Emergency Medicine Fellow
